# MicroRNAs in Human Pituitary Adenomas

**DOI:** 10.1155/2014/435171

**Published:** 2014-12-08

**Authors:** Xu-Hui Li, Elaine Lu Wang, Hai-Meng Zhou, Katsuhiko Yoshimoto, Zhi Rong Qian

**Affiliations:** ^1^Tsinghua-Peking Center for Life Sciences, Tsinghua University, Beijing 100084, China; ^2^Zhejiang Provincial Key Laboratory of Applied Enzymology, Yangtze Delta Region Institute of Tsinghua University, Jiaxing, Zhejiang 314006, China; ^3^Department of Legal Medicine, Kanazawa Medical University, 1-1 Daigaku, Uchinada, Ishikawa 920-0293, Japan; ^4^Department of Pathology, Institute of Health Biosciences, The University of Tokushima Graduate School, Tokushima 770-8503, Japan; ^5^Department of Medical Pharmacology, Institute of Health Biosciences, The University of Tokushima Graduate School, Tokushima 770-8504, Japan; ^6^Center for Molecular Oncologic Pathology, Dana-Farber Cancer Institute, Harvard Medical School, Boston, MA 02215, USA; ^7^Department of Medical Oncology, Dana-Farber Cancer Institute, Harvard Medical School, 450 Brookline Avenue, Room M420, Boston, MA 02215, USA

## Abstract

MicroRNAs (miRNAs) are a class of recently identified noncoding RNAs that regulate gene expression at posttranscriptional level. Due to the large number of genes regulated by miRNAs, miRNAs play important roles in many cellular processes. Emerging evidence indicates that miRNAs are dysregulated in pituitary adenomas, a class of intracranial neoplasms which account for 10–15% of diagnosed brain tumors. Deregulated miRNAs and their targets contribute to pituitary adenomas progression and are associated with cell cycle control, apoptosis, invasion, and pharmacological treatment of pituitary adenomas. To provide an overview of miRNAs dysregulation and functions of these miRNAs in pituitary adenoma progression, we summarize the deregulated miRNAs and their targets to shed more light on their potential as therapeutic targets and novel biomarkers.

## 1. MicroRNAs

MicroRNAs (miRNAs) are a class of recently identified noncoding RNAs. Mature miRNAs are short single-stranded RNA molecules, approximately 19–23 nucleotides in length. The miRNA sequence is encoded in a stem-loop structure in the primary transcript that is cleaved in the nucleus by the ribonuclease III enzyme Drosha to form the precursor miRNA (pre-miRNA). The pre-miRNA is subsequently exported to the cytoplasm by the exportin and then is cleaved by another ribonuclease III enzyme Dicer to form mature miRNA [1–3]. Mature miRNAs can regulate the expression of a large number of genes at the posttranscriptional level. miRNA is partially complementary to the sequence of miRNA recognition elements (MRE) in the 3′ untranslated regions (UTRs) of target mRNAs. The seed sequence with seven nucleotides in miRNA determines the specificity of mRNA targeting, whereas the remaining miRNA sequence is supposed to stabilize the miRNA-target complex [4]. miRNA can inhibit translation of target mRNAs by blocking protein translation machinery or by sequestering the mRNA transcript away from ribosomal interaction. miRNA can also induce target mRNA degradation in a similar way like RNA interference [1, 5]. miRNAs have been identified in a wide range of species, and computational analysis shows that nearly 30% of protein-coding genes can be modulated by miRNAs [6]. In general, miRNAs negatively regulate the expression of their targets. However, it is also reported that miR-369-3p can upregulate the expression of its target, tumor necrosis factor-*α* (TNF-*α*) [7].

miRNAs have been demonstrated to play important roles in many biological processes, such as cell cycle control, proliferation, apoptosis, differentiation, metabolism, hemopoiesis, and development [8]. A rapidly growing body of evidence shows that miRNAs also have comprehensive functions in tumor progression. Some miRNAs may function as oncogenes (also called oncomirs) while some miRNAs are supposed to be tumor suppressors [9]. The importance of miRNAs in cancer is highlighted by the fact that half of all miRNA genes are located in cancer-associated regions or fragile sites, which are frequently altered or deleted in cancer [10]. Many tumor types show unique miRNA signatures; thus, miRNAs may be of use in cancer diagnosis and prognosis [11, 12].

## 2. Pituitary Adenomas

Pituitary adenomas are usually benign intracranial neoplasms, accounting for 10–15% of diagnosed brain tumors [13]. Pituitary adenomas can be derived from a single mutant cell of five differentiated cell types within pituitary gland: somatotropes, lactotropes, corticotropes, thyrotropes, and gonadotropes, which, respectively, secrete growth hormone (GH), prolactin (PRL), adrenocorticotrophic hormone (ACTH), thyroid-stimulating hormone (TSH), and gonadotropins (follicle-stimulating hormone (FSH) and luteinizing hormone (LH)). According to the hormonal activity, pituitary adenomas can be defined as “functioning,” causing endocrine dysfunction such as Cushing's disease in ACTH-secreting pituitary adenomas, acromegaly in GH-secreting pituitary adenomas, galactorrhea and amenorrhea in PRL-secreting pituitary adenomas, and hyperthyroidism in TSH-secreting pituitary adenomas. On the other hand, nonfunctioning pituitary adenomas (NFA) do not give rise to hormone hypersecretion [14].

Pituitary adenomas might be small lesions with slow growth. However, some pituitary adenomas grow rapidly and cause tumor mass effect, the local compressive effect of large pituitary tumors on brain structures and cranial nerves. They can also invade downwards into the paranasal sinuses, laterally into the cavernous sinuses and upwards into the parenchyma of the brain. Occasionally, malignant pituitary carcinomas metastasize to distant locations in the central nervous system, lymph nodes, liver, and other sites throughout the body [13].

In recent years, some reports demonstrated that pituitary adenomas have altered expression files of miRNAs. Nevertheless, the correlation and function of miRNAs and their target genes in pathogenesis of pituitary adenomas remain largely unknown. Only a small number of miRNAs with their target genes in pituitary adenomas have been validated so far. In this review, we summarize recent advances in the study of miRNAs and their validated or potential targets in pituitary adenomas and discuss the future perspectives.

## 3. MicroRNAs in Pituitary Adenomas

### 3.1. Altered miRNA Expression in Pituitary Adenomas

Aberrant expressions of miRNAs have been demonstrated so far (Table 1). miR-15a and miR-16-1 are the first two miRNAs shown to have differential expression in pituitary adenomas. miR-15a and miR-16-1 genes are located at chromosome 13q14, a region which is frequently deleted in pituitary tumors [24]. Previous studies have suggested that the genes in this locus may be responsible for the progression of pituitary adenoma to a more aggressive form [25]. In 2005, miR-15a and miR-16-1 were reported to have lower expression in both GH-secreting and PRL-secreting pituitary adenomas than in normal tissues, and their downregulation was correlated with greater tumor volume and impaired secretion of p43, a potent anticancer cytokine, suggesting that miR-15a and miR-16-1 may function as tumor suppressors and their inactivation may contribute to tumor growth in pituitary adenomas [26]. In another study on ACTH-secreting pituitary tumors, miR-15a and miR-16 were also expressed at a lower level [27], but no association between miRNAs expression and tumor size was observed in this study. This is in accordance with the result of a subsequent report which showed no correlation between downregulation of miR-15a and GH-secreting pituitary tumor size [28]. Mutations in miR-16-1 gene have been reported to be partially responsible for its altered expression in chronic lymphocytic leukemia (CLL) patients [29]. Thus, it is worth exploring whether there are similar mutations in pituitary adenoma patients.

let-7 is one of the first members in the miRNA family. let-7 family members are located at chromosomal regions that are often altered or deleted in human tumors [10]. Downregulation of let-7 has been reported in breast, lung, colon, and others cancers [30–33] and let-7 is considered a tumor suppressor by targeting RAS oncogene [34]. Recently, some studies revealed that high-mobility group A2 (HMGA2) is negatively regulated by the let-7 miRNAs* in vitro* [35, 36]. HMGA2 plays diverse roles in many biological processes such as embryogenesis, differentiation, and neoplastic transformation [37]. Overexpression of HMGA2 is a hallmark of various tumors, including pituitary adenomas, and is associated with highly malignancy [38, 39]. The transgenic mice with overexpressed HMGA2 developed pituitary adenomas, indicating that HMGA2 may be involved in pituitary tumorigenesis [40]. In 2009, Qian et al. reported the clinical significance of HMGA2 overexpression in pituitary adenomas [15]. HMGA2 was frequently upregulated in pituitary adenomas including PRL, ACTH, FSH/LH, or null cell adenomas but relatively rare in GH and mixed GH/PRL adenomas. The authors also reported decreased expression of let-7 in pituitary adenomas. Intriguingly, an inverse correlation between HMGA2 and let-7 was confirmed in this study. HMGA2 overexpression and the decrease of let-7 were significantly correlated with tumor proliferation, growth, invasion, and tumor grade, which lead to a hypothesis that let-7 may also function as a tumor suppressor in pituitary adenomas by targeting HMGA2. Decreased expression of let-7a in pituitary adenomas was also reported in other studies [27, 41], suggesting the general downregulation of let-7 in pituitary adenomas. On the other hand, some other miRNAs such as miR-98 can also regulate HMGA2 expression [42], indicating that HMGA2 may have multiple miRNAs regulators. During pituitary development, let-7b/c was proposed to operate with the RNA-binding protein KSRP in a negative feedback loop, in which KSRP induces the maturation of let-7b/c, and let-7b/c posttranscriptionally downregulates the expression of KSRP itself [43].

As pituitary adenomas can be derived from differentiated cell types within pituitary gland, different subtypes of pituitary adenomas could display distinct miRNA profiles, and these specific profiles might be useful to distinguish pituitary adenoma subtypes. In 2007, a list of thirty miRNAs differentially expressed in pituitary adenomas was generated by microarray [41]. Seven miRNAs were upregulated and twenty-three were downregulated. The most representative ones were miR-212, miR-026a, miR-150, miR-152, miR-191, and miR-192, which were upregulated in pituitary adenomas, while miR-024-1 and miR-098 were downregulated in tumor samples. Twenty-nine miRNAs were identified to be able to predict pituitary adenoma histotype (ACTH-, GH-, PRL-secreting adenomas, and NFA). For the limit of sample numbers, the authors only analyzed the association of deregulated miRNAs and tumor diameter in the NFA group. Five miRNAs were upregulated (miR-140, miR-099a, miR-099b, miR-030b, and miR-030c) and only one (miR-138-2) was downregulated in macroadenomas compared to microadenomas.

In 2009, Amaral et al. investigated the differential expression of some miRNAs in ACTH-secreting pituitary tumors. In addition to the decrease of let-7a, miR-15a, and miR-16, they also found underexpression of miR-21, miR-141, miR-143, miR-145, and miR-150 in ACTH-secreting pituitary adenomas compared with normal pituitary tissues [27]. Among these miRNAs, downregulation of miR-141 has been reported in gastric cancer [44] and renal cell carcinoma [45]. miR-143 expression was decreased in human lung and colorectal cancers [46, 47] and was reported to inhibit KRAS translation in colorectal cancer cell [48]. miR-145 was downregulated in human breast, lung, and colorectal cancers [30, 46, 47, 49]. miR-145 could regulate the expression of various targets in different tumors: FSCN1 in esophageal squamous cell carcinoma [50], OCT4, EGFR, and NUDT1 in lung adenocarcinoma [51, 52], and FLI1 in colon cancer [53]. miR-143/145 cluster is a target of Jagged-1/Notch signaling in vascular smooth muscle cells [54]. miR-150 was overexpressed in hematopoietic progenitor/stem cells [55] and was demonstrated to target NOTCH3 in human T-cell development in a recent study [56].

Studies were conducted with the aim of investigating the aberrant expression of miRNAs in GH-secreting pituitary adenomas. In 2010, Mao et al. identified totally fifty-two miRNAs to be differentially expressed in GH-secreting pituitary adenomas. Nine of these miRNAs had altered expression between macro- and microadenomas. miR-184, miR-524-5p, miR-629, and miR-766 were upregulated, while miR-124, miR-222, miR-32, miR-744, and miR-765 were downregulated [28]. In 2012, another set of miRNAs were identified to be differentially expressed in GH-secreting pituitary adenomas [19]. Eighteen miRNAs, including miR-34b, miR-326, miR-432, miR-548c-3p, miR-570, and miR-603, were drastically and constantly downregulated in GH adenomas, whereas only miR-320 was significantly upregulated. miR-34b and miR-548c-3p were demonstrated to regulate both HMGA1 and HMGA2 expression, whereas miR-326, miR-432, and miR-570 target HMGA2 only. miR-326 and miR-603 could decrease the expression of the E2 transcription factor 1, E2F1. Besides, miR-107 was found to be overexpressed in GH-secreting and nonfunctioning pituitary adenomas and inhibited the expression of pituitary tumor suppressor gene aryl hydrocarbon receptor-interacting protein (AIP) [20]. Recently, Palumbo et al. identified 17 miRNAs which were differentially expressed in GH-secreting pituitary tumors. Specifically, five miRNAs (miR-26b, miR-26a, miR-212, miR-107, and miR-103) were upregulated and twelve miRNAs (miR-125b, miR-141, miR-144, miR-164, miR-145, miR-143, miR-15b, miR-16, miR-186, let-7b, let-7a3, and miR-128) were downregulated. miR-26b and miR-128 controlled pituitary cell properties through regulation of their direct targets, PTEN, and BMI1, respectively [18]. miR-26b also targeted Lef-1 and increased Pit-1 expression in GH3 cells [57].

miRNAs are also dysregulated in nonfunctioning pituitary adenomas (NFA). In 2011, Butz et al. analyzed miRNAs expression in NFA and the signaling pathways altered in these pituitary tumors [22]. Expressions of Smad3, Smad6, Smad9, MEG, and DLK1 were significantly decreased in NFA. Through pathway analysis and in silico target prediction, a specific subset of miRNAs was identified that may potentially downregulate TGF-*β* signaling pathway in NFA. Five miRNAs predicted to target Smad3 (miR-135a, miR-140-5p, miR-582-3p, miR-582-5p, and miR-938) were overexpressed, of which miR-140-5p has already been validated to target Smad3 directly [58]. In addition, an inverse correlation between tumor size and the expression of eighteen miRNAs was observed. Six miRNAs of them (miR-450b-5p, miR-424, miR-503, miR-542-3p, miR-629, and miR-214) were significantly underexpressed, while one miRNA (miR-592) was significantly overexpressed in NFA compared to normal pituitary tissues. In another study, miR-124a was the most upregulated miRNA, and miR-31 was the most downregulated miRNA in nonfunctioning pituitary adenomas [59].

In gonadotropin-secreting pituitary adenomas, a study demonstrated that miR-10b was upregulated and miR-503 was downregulated [59]. Furthermore, the integration and coordination of hormones and pituitary cells are important for the regulatory function of pituitary tissues. Gonadotropin-Releasing Hormone (GnRH) acts on pituitary gonadotropes to stimulate LH and FSH synthesis and secretion. GnRH induces expressions of miR-132 and miR-212 in L*β*T2 pituitary gonadotrope cells to regulate cellular morphology and migration. The p250RhoGAP protein is a downstream target of miR132/212 and its downregulation is involved in the morphological change and migration altered by GnRH [60].

### 3.2. Functional Association of miRNAs in Pituitary Tumorigenesis and Progression

#### 3.2.1. miRNAs and Cell Cycle in Pituitary Adenomas

**Figure 1:**
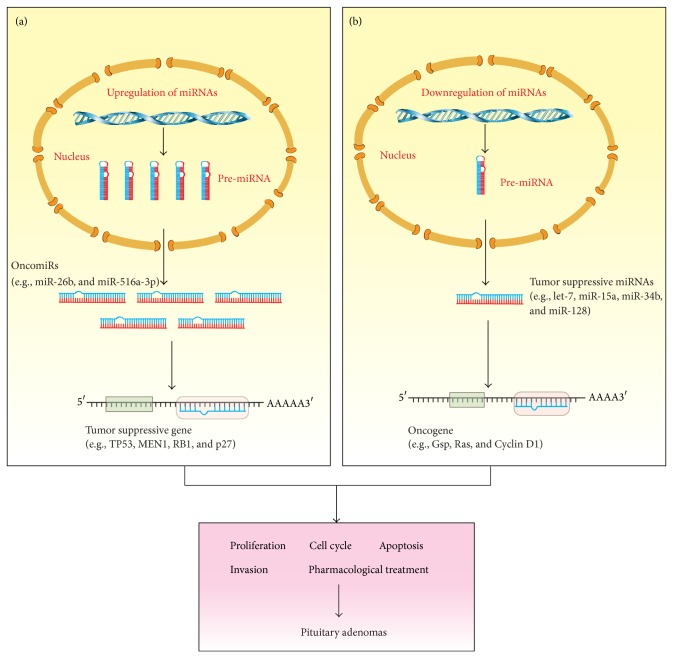
Functions of miRNAs as oncogenic and tumor suppressive genes in pituitary adenomas. (a) Upregulation of oncogenic miRNAs (oncomiRs) in pituitary adenomas results in suppression of their target tumor suppressor genes (e.g., TP53, MEN1, and RB1). (b) Downregulation of tumor suppressive miRNAs results in upregulation of their target oncogenes (e.g., Gsp, Ras, and Cyclin D1). The consequence of oncomiRs and tumor suppressive miRNAs regulation in pituitary adenomas might involve aberrant proliferation, cell cycle control, apoptosis, and invasiveness.

It is well known that the dysfunction of cell cycle control is a critical step in initiation and progression of human cancers. Some oncoproteins or tumor suppressors play important roles in cell cycle control by interacting with critical cell cycle regulators, such as cyclin, cyclin-dependent-kinase (CDK), or cell cycle inhibitors. During tumor progression, the genes involved in cell cycle control often have aberrant expression, resulting in unlimited tumor cell growth [61]. Some reports suggested that the deregulated miRNAs might also regulate cell cycle of pituitary adenomas at the post-transcriptional level (Figure 1).

Wee1 was described as a tumor suppressor to inhibit cell cycle. Wee1 phosphorylates Cdk1 and inhibits its activity to block cell cycle in G2/M checkpoint [62]. Wee1 was downregulated in GH-secreting and NFA pituitary adenomas. miR-128a, miR-155, and miR-516a-3p target 3′-UTR of Wee1, and exogenous overexpression of these miRNAs inhibited Wee1 expression [21]. miR-128a is a brain-enriched miRNA and was reported to be decreased in pituitary adenomas [41]. Its ectopic overexpression reduced neuroblastoma cell motility and invasiveness [63], suggesting its tumor suppressive role. miR-155 was reported as an oncomir in both hemopoietic malignancies and solid tumors [64]. miR-516a-3p was involved in glioblastoma development [65] and was associated with progression of breast cancer [66]. These miRNAs may take part in the regulation of cell cycle in pituitary adenomas together with other related miRNAs.

HMGA2 is associated with the E1A-regulated transcriptional repressor p120 (E4F), interfering with p120 (E4F) binding to the cyclin A promoter. Ectopic expression of HMGA2 resulted in the activation of cyclin A promoter and induction of endogenous cyclin A expression. Moreover, chromatin immunoprecipitation experiments showed that HMGA2 was associated with cyclin A promoter only when the gene was transcriptionally activated. These data indicate cyclin A as a cellular target of HMGA2 and, for the first time, lead to a mechanism of HMGA2-dependent cell cycle regulation [67]. Thus, let-7, as a regulator of HMGA2, may exert its effects in cell cycle control of pituitary adenomas by targeting HMGA2. miR-23b and miR-130b, which were reduced in GH, gonadotroph, and NFPA adenomas, were demonstrated to target HMGA2 and cyclin A2, respectively. Overexpression of miR-23b and miR-130b arrested the cells in the G1 and G2 phase of the cell cycle [16].

Recently, a study revealed that miR-15a and miR-16-1 cluster could modulate prostate cancer by targeting multiple genes, including cyclin D1 [68]. Regarding the deregulation in pituitary adenomas, miR-15a and miR-16-1 may exert their roles as tumor suppressors by regulating cell cycle. Expression of miR-126 and miR-381 was decreased in GH-secreting pituitary adenomas [28]. Previous study has shown that mir-126 could modulate phosphatidylinositol 3-kinase (PI3K) signaling by limiting the PI3K regulatory subunit beta (p85b). Loss of miR-126 would eliminate the check point and increase PI3K signaling, which facilitate tumor growth during colon carcinogenesis [69]. miR-145 was downregulated in GH-secreting pituitary adenomas [28], which is in line with the results in 11 samples of cortitropinomas [27]. The potential targets of miR-145 include myc, kras, fos, yes, fli, cyclin D2, and MAPK transduction proteins [30], indicating that miR-145 might function in cell cycle control by targeting multiple genes. miR-503 was highly expressed in NFA and had a negative correlation with tumor size [22]. miR-503 has been validated to directly target cyclin D1 and is thought to be a tumor suppressor [70]. Furthermore, an important potential target of miR-503 is the cell cycle regulator CDC25.

miR-26b and miR-128 were found to directly regulate PTEN and BMI1, respectively. Moreover, miR-128 regulated PTEN expression and Akt activity in the pituitary tumor cells by interfering with the binding of BMI1 to PTEN promoter [18]. Since PTEN-Akt pathway plays important roles in cell cycle control, miR-26b and miR-128 might regulate cell cycle through PTEN-Akt pathway [71]. Moreover, miR-26a was also overexpressed in ACTH-secreting pituitary adenomas and plays an important role in cell cycle control by modulating protein kinase C delta [17].

#### 3.2.2. miRNAs and Apoptosis in Pituitary Adenomas

Apoptosis, the process of programmed cell death, is an important barrier for tumor cells. During malignant transformation and tumor progression, tumor cells have to escape this regulated cell death to obtain an advantage in growth and expansion. At the early stage of apoptosis, cells receive death signals, and then the “apoptotic trigger” is controlled by pro- or antiapoptotic members of B-cell lymphoma 2 (Bcl-2) family and other regulatory proteins [72]. Accumulating evidence have shown that miRNAs can regulate cancer cell apoptosis by targeting Bcl-2 family or other apoptosis regulators (Figure 1).

miR-15a and miR-16-1 were demonstrated to induce apoptosis by targeting Bcl-2 in CLL [73]. Bcl-2 is a founding member of the Bcl-2 family, a family of antiapoptotic proteins governing mitochondrial death signaling. Bcl-2 is frequently overexpressed in many types of human cancers, including carcinomas, lymphomas, and leukemias [74]. In CLL, some other apoptosis related genes were identified to be targets of miR-15a and miR-16-1 cluster, such as MCL1, which could enhance cell survival by inhibiting apoptosis. Therefore, it is possible that, in pituitary adenomas, miR-15a and miR-16-1 influence apoptosis by targeting multiple antiapoptotic genes. Besides, miR-214 and miR-629, two miRNAs overexpressed in NFA and negatively correlated with tumor size, also potentially target Bcl2 [22].

miR-21 was differentially expressed in ACTH-secreting pituitary adenomas compared with normal pituitary tissues [27]. miR-21 has been identified to be upregulated in human breast, lung, colorectal and other cancers [30, 46, 49, 75]. Suppression of miR-21 by antisense oligonucleotides or miR-21 knockdown was associated with increased apoptotic activity and inhibition of tumor cell growth, probably by downregulating the target tumor suppressor genes [76]. miR-21 may exert its function in apoptosis by targeting tumor suppressor Pdcd4 [77] and PTEN [78]. Overexpression of PDCD4 was able to result in apoptotic death [79], and PTEN can induce apoptosis through phosphoinositol-3-kinase/Akt dependent and independent pathways [80]. miR-21 is upregulated both* in vitro* and* in vivo* by oncogenic Ras [81].

miR-212 is strongly upregulated in pituitary adenomas [41]. Putative targets of miR-212 include death effector domain-containing protein (DEDD), a protein involved in apoptotic signaling [82], as well as other proteins participating in apoptosis. miR184 was markedly upregulated in GH-secreting pituitary adenomas and was correlated with tumor diameter [28]. Contrary to that, another study reported that ectopic overexpression of miR-184 resulted in increased apoptosis [83]. Study of Cheng et al. suggested that the upregulated miR-150, miR-152, miR-191, and miR-192 may also be involved in apoptosis [84].

miR-26b was found to be upregulated in GH-secreting pituitary tumors and directly regulate PTEN. Therefore, miR-26b is able to regulate apoptosis through PTEN-Akt pathway. miR-200c, which has been characterized as a tumor suppressor or oncogene in different cancers, also inhibited apoptosis in pituitary adenoma cells by targeting the PTEN/Akt signaling pathway [23]. Intriguingly, a novel marine drug, SZ-685C that was isolated from the secondary metabolites of a mangrove endophytic fungus was reported to induce apoptosis of MMQ pituitary tumor cells by downregulating miR-200c [85].

TGF-*β* has been shown to inhibit proliferation and induce apoptosis in HP75 cells, a cell line derived from a clinically NFA [86]. Thereby, the miRNAs targeting TGF-*β* signaling (miR-135a, miR-140-5p, miR-582-3p, miR-582-5p, and miR-938) may have effects in apoptosis [22]. However, as TGF-*β* can also promote cancer cell invasion by inducing Epithelial-Mesenchymal Transition (EMT) [87], it is rational to conclude that miRNAs targeting TGF-*β* pathway may suppress invasion and metastasis by blocking EMT, as miR-300 does in human epithelial cancer [88]. Therefore, miRNAs that regulate TGF-*β* pathway play controversial roles in tumor initiation and progression. Deregulation of BMI1 has been revealed to affect apoptosis; thus, miR-128, which was downregulated in GH-secreting pituitary tumors, could also affect apoptosis by directly regulating BMI1 [18]. These data together lead to the hypothesis that many miRNAs may function in a network to regulate apoptosis in pituitary adenomas.

#### 3.2.3. miRNAs and Invasion or Metastasis of Pituitary Adenomas

Invasion and metastasis are critical lethal factors for malignant cancer. Although invasion and metastasis are rare in pituitary tumors, studies provide some clues of miRNAs' function in pituitary tumor invasion and metastasis (Figure 1).

Significant correlation between HMGA2 overexpression and tumor cell invasion has been detected in breast cancer and gastric cancer [89, 90]. In oral squamous cell carcinomas, strong staining of HMGA2 and loss of E-cadherin expression were observed at the invasive front of tumor [91]. Previous studies also demonstrated that tumor-specific downregulation of E-cadherin and H-cadherin was related to invasiveness of pituitary adenoma [92]. HMGA2 may be involved in tumor cell invasion due to its association with epithelial-mesenchymal transition that facilitates tumor cell invasion. Since let-7 regulates HMGA2 expression in pituitary adenomas, let-7 may also take a role in pituitary adenoma invasion. In Amaral et al.'s study, although no association between miRNAs expression and tumor size was observed, the patients with ACTH-secreting pituitary tumors expressing reduced miR-141 had more chance of remission after transsphenoidal surgery, suggesting that miR-141 may regulate pituitary genes involved in tumor growth and local invasion [27]. PTTG protein 1 is a target of both miR-126 and miR-381, which were downregulated in GH-secreting pituitary adenomas [28]. PTTG is overexpressed in most pituitary adenomas and is involved in tumor invasion [93]. Therefore, miR-126 and miR-381 might regulate pituitary adenoma invasion by targeting PTTG.

Aggressive pituitary adenomas and carcinomas frequently have a deletion in regions near the RB gene [94, 95]. In 2010, Stilling et al. investigated the expression of miRNAs in pituitary carcinomas [96]. In one case, ACTH carcinoma had metastatic carcinomas in three sites. More miRNAs were deregulated between pituitary adenomas and normal pituitaries compared to carcinomas and normal pituitaries. In pituitary carcinomas compared to ACTH adenomas, miR-122 and miR-493 were upregulated, and, in all three metastatic sites of ACTH carcinomas, miR-122 expression was markedly increased.

Recently, Palumbo et al. identified miR-26b to be upregulated and miR-128 to be downregulated in GH-secreting pituitary tumors [18]. Inhibition of miR-26b and overexpression of miR-128 suppressed colony formation and invasiveness of pituitary tumor cells. Interestingly, the inhibition of miR-26b and overexpression of miR-128 had a synergistic effect on suppressing the tumorigenicity and invasiveness of pituitary tumors. Since deregulation of PTEN and BMI1 correlates with the invasive and metastatic phenotype of several human cancer types [97, 98], it is possible that miR-26b and miR-128 regulate invasiveness of pituitary tumor cells by directly targeting PTEN and BMI1, respectively. Although metastatic pituitary carcinomas are rare, these data suggest that altered expression of miRNAs may provide diagnostic information to distinguish pituitary adenomas and carcinomas before they metastasize.

#### 3.2.4. miRNAs and Pharmacological Treatment of Pituitary Adenomas

The symptoms of mass effect and hormonal hypersecretion caused by pituitary adenomas could be reversed by surgical resection or debulking of the adenoma, radiotherapy, or medical treatment. Medical treatment is the primary choice for prolactinomas and the secondary option for acromegaly, Cushing's disease, gonadotropin-secreting tumours, and TSH-secreting adenomas [99]. Some studies provide evidence that miRNAs were differentially expressed before and after pharmacological treatment, and the altered miRNA profile could provide useful information of responsiveness of pituitary adenomas patients to pharmacological treatment (Figure 1).

In 2007, a microarray was carried out to analyze the miRNA profiles in pituitary adenomas and normal pituitary samples. To elucidate whether miRNAs profile is altered by pharmacological treatment, differentially expressed miRNAs were identified in NFA from patients with pharmacological treatment or patients without treatment [41]. Six miRNAs were found to be differentially expressed: miR-29b, miR-29c, and miR-200a were upregulated, while miR-134, miR-148, and miR-155 were downregulated after treatment. Cluster analysis showed clear distinction between pharmacological treated and nontreated NFA. Thus, the miRNA expression could differentiate treated patient samples from nontreated patient samples.

In 2010, another study aimed to identify altered expression of miRNAs in GH-secreting pituitary adenomas [28]. Fifteen pituitary adenomas patients were treated with lanreotide for four months before surgery, while six patients did not receive any presurgical medical treatments. Patients with >50% reduction of GH secretion after lanreotide treatment were considered somatostatin analogs (SSA) responders, while patients with <50% GH secretion were considered SSA nonresponders [100]. Thirteen miRNAs were differentially expressed between GH-secreting pituitary adenomas from patients with lanreotide treatment and those without treatment. Eight miRNAs (miR-183, miR-193a-5p, miR-222, miR-516b, miR-524-5p, miR-601, and miR-629, 99b) were upregulated and five miRNAs (miR-124, miR-32, miR-574-5p, miR-744, and miR-96) were downregulated. Moreover, seven miRNAs were differentially expressed between SSA responders and SSA nonresponders. Putative targets of these miRNAs are mainly IGFBP family members, IGFALS, SCP1, and matrix metalloproteinase-9.

## 4. Conclusion and Future Perspectives

Accumulating evidence demonstrates that a large number of miRNAs have altered expression in pituitary adenomas, and these miRNAs may play important roles in tumor progression by targeting multiple genes. The molecular mechanism of the regulation of miRNAs in pituitary adenomas is still a mystery. Some proofs indicate that genetic or epigenetic alterations may contribute to the deregulated expression of miRNAs. For example, mutations in the miR-16-1 gene have been reported to be partially responsible for its aberrant expression in CLL patients [29], and expressions of miR-124 and miR-203 are decreased because of CpG methylation [101]. Some miRNAs have been demonstrated to target multiple genes, indicating that they may have different roles in pituitary tumors. On the other hand, a gene involved in pituitary adenomas progression can be modulated by more than one miRNA. Therefore, the miRNAs and their targets could regulate pituitary adenomas progression in a complex network.

Advances in the technology to investigate miRNAs make it easier and faster to explore more exactly the roles of miRNAs in pituitary adenomas. As some miRNAs signatures can be used to distinguish pituitary adenomas and normal pituitaries and even subtypes of pituitary tumors, it is also possible to develop miRNA based diagnosis and therapies of pituitary adenomas. The knowledge of pituitary pathogenesis is still limited. Continuing study on miRNAs and their targets will shed more light on mechanisms of pituitary adenomas.

## Figures and Tables

**Table 1:** MicroRNAs and their target genes in human pituitary adenomas.

miRNA	Upregulated or downregulated	Target genes	Tumor type	Reference
let-7	Downregulated	*HMGA2 *	PRL, ACTH FSH/LH	[15]
miR-23b	Downregulated	*HMGA2 *	GH, NFA FSH/LH	[16]
miR-26a	Upregulated	*PRKCD *	ACTH	[17]
miR-26b	Upregulated	*PTEN *	GH	[18]
miR-34b	Downregulated	*HMGA1, HMGA2 *	GH	[19]
miR-107	Upregulated	*AIP *	GH, NFA	[20]
miR-128	Downregulated	*BMI1 *	GH	[18]
miR-128a	Upregulated	*Wee1 *	NFA	[21]
miR-130b	Downregulated	*CCNA2 *	GH, NFA FSH/LH	[16]
miR-140-5p	Upregulated	*Smad3 *	NFA	[22]
miR-155	Upregulated	*Wee1 *	NFA	[21]
miR-200c	Upregulated	*PTEN *	PRL	[23]
miR-326	Downregulated	*HMGA2, E2F1 *	GH	[19]
miR-432	Downregulated	*HMGA2 *	GH	[19]
miR-516a-3p	Upregulated	*Wee1 *	NFA	[21]
miR-548c-3p	Downregulated	*HMGA1, HMGA2 *	GH	[19]
miR-570	Downregulated	*HMGA2 *	GH	[19]
miR-603	Downregulated	*E2F1 *	GH	[19]
